# Dataset of pollutants in organic and non-organic food

**DOI:** 10.1016/j.dib.2022.108295

**Published:** 2022-05-19

**Authors:** Gilles-Eric Seralini, Jérôme Douzelet, Gérald Jungers

**Affiliations:** Network on Risks, Quality and Sustainable Environment, University of Caen Normandy MRSH and Faculty of Sciences, Esplanade de la Paix, Caen 14032 CEDEX, France

**Keywords:** Pesticides, Polycyclic aromatic hydrocarbons, Heavy metals, Organic food, Non-organic food, Sausages, Cheeses, PAHs, Polycyclic Aromatic Hydrocarbons, C, cheeses, S, sausages, Sp, Spain, Fr, France, Ge, Germany, O, Organic, NO, Non-organic, Sm, Smoked, NSm, Non-smoked

## Abstract

Organic and non-organic equivalent corresponding type of foods were assessed for pollutants, polycyclic aromatic hydrocarbons (PAHs), pesticides and heavy metals. In total, 35 samples of sausages and cheeses were tested from three western European countries, Spain, France, and Germany. These samples were chosen because they are meat and milk products presenting some bioaccumulation capacity. Petroleum residues were demonstrated in pesticide formulants. These include polycyclic aromatic hydrocarbons (PAHs) and metals. We measured in these human foods 800 pesticides, 24 PAHs, using accredited methods, as well as eleven metals and elements. Pesticides’ measurements were performed either by gas or liquid chromatography, followed by mass spectrometry, according to regulatory standard methods, that were measured over the threshold of 10 μg/kg for this reason. This could be insufficient to detect all toxic contaminations. Metals were analysed by high frequency induced plasma emission spectrometry and mass spectrometry after pressurized digestion. PAHs were assessed by gas and/or high-performance liquid chromatography with reverse phase polarity and if necessary double mass spectrometry. The raw data are summarized in Tables 1 and 2. The dataset can be reused for statistical analyses by the scientific community to evidence food pollution and to be compared with other measurements.

## Specifications Table

**Table:** 

Subject	Agricultural Sciences – Food Science: Food Chemistry
Specific subject area	Measurement of human food pollution by pesticides, heavy metals and polycyclic aromatic hydrocarbons, in non-organic and corresponding organic sausages and cheeses.
Type of data	2 large tables as specified.
How the data were acquired	Pesticide measurements were performed either by gas or liquid chromatography, followed by mass spectrometry, according to regulatory methods adapted for food. Metals were analysed mostly by high frequency induced plasma emission spectrometry and mass spectrometry. PAHs were assessed by gas and/or high-performance liquid chromatography with reverse phase polarity and if necessary double mass spectrometry.
Data format - raw	Raw original data, not published elsewhere, are indicated in the 2 tables.
Description of data collection	The exact names of commercialized products, locations, names of producers, distributors, lot numbers were indicated. The certificates of foods, if any, are given. On the other Table 2, in each food, raw data of metals levels are given, as well as 24 polycyclic aromatic hydrocarbons levels, totals, and pesticides found. No inclusion / exclusion criteria were applied. No normalization was applied except for regulatory thresholds.
Data source location	The locations, countries and producers were specified and sold in many stores in France, Spain and Germany, other specificities do not apply.
Data accessibility	All data referred to in your data article were made publicly available prior to publication. All raw data are shared via a data repository, these are not secondary data. Repository name: Mendeley Data Title: Dataset of pollutants in organic and non—organic food-corr Direct URL to data: Mendeley Data, V1, doi: 10.17632/rmycjhph5t.1
Related research article	Published article related to this dataset [1]: Detection of Pollutants in Organic and Non-Organic Food: Are PAHs Coming from Pesticides? Gilles-Eric Seralini*, Jérôme Douzelet, Gérald Jungers Food Nutr J 7: 238. https://doi.org/10.29011/2575–7091.100238

## Value of the Data

• These data are important to be an element for comparison with previous and further studies, for calculations and to add in other datasets. This corresponds to what we have performed previously [2].
• Other researchers or scientists, and laboratories analyzing food pollutants in general, as well as the public and the media can benefit from these data.
• These data can be reused for statistical studies and more general calculations, to expand knowledge of food composition in general.

## 1 Data Description

We provide here the exact nature of 35 food samples used in this study (Sausages S and Cheeses C). Their origin by country is detailed (Spain Sp, France Fr and Germany Ge), as well as if these are Smoked (Sm) or Non Smoked (NSm), Organic (O), with the name of the Certificate, or NO non-organic. Names and nature of products are indicated, producers, distributors, lot numbers, certificates. In comparison to the related-research article where these data are not indicated [1], some samples have been renumbered: 32 became 33 in Table 1, 33 became 34, 34 became 35 and 35 became 32 to group by the end sausages and cheeses.

The levels in each of these foods (raw data) are indicated for metals, polycyclic aromatic hydrocarbons (PAHs) and pesticides. This was not in the original article.

## 2 Experimental Design, Materials, and Methods

### 2.1 Food

Equivalent organic and non-organic sausages and cheeses were collected in stores in France, Germany, and Spain, and freshly analyzed. The full list appears in the raw data (Table 1). The sampling had to be rapid (a few days) as well as the transport before the assays to optimize the analyses. There is a possibility of bacteriological damage and chemical interactions with packaging. We compared the results in two different laboratories to check the reliability and reproducibility of the results and to validate the following methods.

### 2.2 Pesticides

The extraction was performed by QuEChERS TS/EN method 15662 adapted for foods, publicly available. It is a multimethod for the determination of pesticide residues by gas and liquid chromatography analysis after extraction/partition with acetonitrile and purification by dispersive solid phase extraction - QuEChERS modular method. The method has been the subject of an interlaboratory test involving a large number of commodity/pesticide combinations. Precision data are summarized in FD CEN/TR 17063. Calibration guidelines are given in prCEN/TS 17061 available in each european langage on internet.

The screening for 800 pesticides included glyphosate, glufosinate and AMPA. It was performed by gas and liquid chromatography, followed by mass spectrometry (using either one of these methods or both) [3], according to regulatory methods adapted for food, especially through § 64 LFGB L 00.00-34: 2010-09, or 00.00-113: 2015-03, fully and publicly available on internet.

### 2.3 Metals

A total of 11 metals and elements (Ag, As, Cd, Co, Cu, Fe, Ni, Pb, Si, Ti, Zn) were analysed by adapted regulatory methods for food, mostly by high frequency induced plasma emission spectrometry and mass spectrometry (ICP-MS) [4] after pressurized digestion, called norms AFNOR, ME48, DIN EN ISO 15763, EN ISO 17294-2-E29 publicly available on internet.

### 2.4 Polycyclic Aromatic Hydrocarbons (PAHs)

In total, 24 main PAHs (Table 2) were assessed by gas and/or high-performance liquid chromatography with reverse phase polarity and if necessary double mass spectrometry (GC/MS/MS) [5], according to norm EU 208/2005 or an adapted version of NF EN ISO 15302 or 15753 with GC/MS ISO N842 CD21035 modified publicly available on internet.

## Ethics Statements

The work did not involve human subjects, nor animal experiments, and no data collected from social media platforms; thus, only the general ethical statements for independent and correct science are respected.

## CRediT authorship contribution statement

**Gilles-Eric Seralini:** Conceptualization, Validation, Methodology, Formal analysis, Investigation, Resources, Writing – review & editing, Supervision. **Jérôme Douzelet:** Conceptualization, Validation, Resources, Writing – review & editing. **Gérald Jungers:** Methodology, Validation, Formal analysis, Writing – review & editing, Visualization.

## Declaration of Competing Interest

The authors declare that they have no known competing financial interests or personal relationships that could have appeared to influence the work reported in this paper.

## Data Availability

Dataset of pollutants in organic and non-organic food-corr (original data) (Mendeley Data).
